# Leucine-rich alpha 2 glycoprotein promotes Th17 differentiation and collagen-induced arthritis in mice through enhancement of TGF-β-Smad2 signaling in naïve helper T cells

**DOI:** 10.1186/s13075-017-1349-2

**Published:** 2017-06-14

**Authors:** Hayato Urushima, Minoru Fujimoto, Takashi Mishima, Tomoharu Ohkawara, Hiromi Honda, Hyun Lee, Hirohisa Kawahata, Satoshi Serada, Tetsuji Naka

**Affiliations:** 1 Laboratory of Immune Signal, National Institutes of Biomedical Innovation, Health and Nutrition, 7-6-8 Saito-Asagi, Ibaraki, Osaka 567-0085 Japan; 2 Department of Medical Technology, Morinomiya University of medical science, Osaka, Japan; 3 Center for Intractable Immune Disease, Kochi Medical School, Kochi University, Kochi, Japan

**Keywords:** Leucine rich alpha2 glycoprotein, TGF-β, Smad2, IL-6 receptor, Th17

## Abstract

**Background:**

Leucine-rich alpha 2 glycoprotein (LRG) has been identified as a serum protein elevated in patients with active rheumatoid arthritis (RA). Although the function of LRG is ill-defined, LRG binds with transforming growth factor (TGF)-β and enhances Smad2 phosphorylation. Considering that the imbalance between T helper 17 (Th17) cells and regulatory T cells (Treg) plays important roles in the pathogenesis of RA, LRG may affect arthritic pathology by enhancing the TGF-β-Smad2 pathway that is pivotal for both Treg and Th17 differentiation. The purpose of this study was to explore the contribution of LRG to the pathogenesis of arthritis, with a focus on the role of LRG in T cell differentiation.

**Methods:**

The differentiation of CD4 T cells and the development of collagen-induced arthritis (CIA) were examined in wild-type mice and LRG knockout (KO) mice. To examine the influence of LRG on T cell differentiation, naïve CD4 T cells were isolated from LRG KO mice and cultured under Treg- or Th17-polarization condition in the absence or presence of recombinant LRG.

**Results:**

In the CIA model, LRG deficiency led to ameliorated arthritis and reduced Th17 differentiation with no influence on Treg differentiation. By addition of recombinant LRG, the expression of IL-6 receptor (IL-6R) was enhanced through TGF-β-Smad2 signaling. In LRG KO mice, the IL-6R expression and IL-6-STAT3 signaling was attenuated in naïve CD4 T cells, compared to wild-type mice.

**Conclusions:**

Our findings suggest that LRG upregulates IL-6R expression in naïve CD4 T cells by the enhancement of TGF-β-smad2 pathway and promote Th17 differentiation and arthritis development.

## Background

By a proteomic approach, we previously identified leucine-rich alpha 2 glycoprotein (LRG) as a serum protein that is elevated in patients with active rheumatoid arthritis (RA) [1]. There was stronger correlation between serum LRG and the 28-joint disease activity score (DAS28) in patients with RA than there was with C-reactive protein, suggesting that LRG is a promising biomarker of RA that reflects disease activity quantitatively and objectively. Since LRG contains repetitive leucine-rich sequences known to be involved in protein-protein interactions and signal transduction [2], elevated LRG may have a role in regulating the action of other molecules and/or signal transduction. Recently, other studies [3] and our own study [4] have shown that LRG binds with transforming growth factor (TGF)-β and/or its receptors and modulates downstream pathways of TGF-β signaling. Moreover, pathophysiological functions of LRG have been elucidated in several disease models including pathological ocular angiogenesis [3], myocardial infarction [5] and cancer [4, 6]. However, the role of LRG in autoimmune arthritis is still unclear.

TGF-β has a reciprocal function in differentiation of both T helper 17 cells (Th17) and regulatory T cells (Treg). Namely, by the stimulation of TGF-β alone, naïve T cells differentiate into “anti-inflammatory” Treg [7, 8]. However, in conjunction with IL-6, TGF-β promotes the differentiation into “pro-inflammatory” Th17 cells [9, 10]. Several lines of evidence indicate that the imbalance between Th17 and Treg plays important roles in the pathogenesis of RA. For instance, peripheral blood Th17 frequencies and Th17-related cytokines such as IL-17 and tumor necrosis factor (TNF)-α were significantly increased in RA patients, while Treg frequencies were decreased [11]. Moreover, elevated levels of Th17 cells in the circulation were associated with disease activity in RA [12] and IL-17 expression levels correlated with poor prognosis and the severity of joint destruction [13, 14].

We previously showed that LRG can enhance TGF-β-Smad2 signaling in several cell lines. Because the importance of TGF-β-Smad2 signaling in the differentiation of helper T cells is well-documented, LRG may affect immune homeostasis by enhancing this pathway in CD4 T cells. However, the exact role of TGF-β-Smad2 signaling in CD4 T cells is still elusive and is conceivably context-dependent. Lack of Smad2 in T cells reduces TGF-β-induced upregulation of forkhead box p3 (Foxp3), a master regulator of Treg differentiation [7, 15]. On the other hand, T cell-specific Smad2 deficiency causes a defect in the differentiation of Th17 in vitro and in vivo [15–17]. Thus, even if LRG enhances Smad2 activation in T cells, it needs to be determined whether LRG exhibits an anti-inflammatory function or pro-inflammatory action.

In this study, by using collagen induced arthritis (CIA), a mouse model of rheumatoid arthritis, we aimed to elucidate the involvement of LRG in the pathogenesis of RA, especially focusing on T lymphocyte differentiation.

## Methods

### Generation of LRG-deficient mice

We generated LRG knockout (LRG KO) mice on a C57BL/6 background as previously described [4]. Wild-type (WT) and LRG KO mice were maintained by in-house breeding.

### CIA

CIA was performed as previously described [18]. Briefly, complete Freund’s adjuvant was prepared by grinding 100 mg heat-killed *Mycobacterium tuberculosis* (H37Ra; Difco Laboratories, Detroit, MI, USA) in 20 mL of incomplete Freund’s adjuvant (Sigma, Tokyo, Japan). Chicken type-2 collagen (Sigma) was dissolved in 10 mM acetic acid overnight at 4 °c. An emulsion was formed by combining 2 mg/mL chicken type-2 collagen in acetic acid with an equal volume of complete Freund’s adjuvant (5 mg/mL). Ten-week-old WT or LRG KO mice were injected intra-dermally at several sites into the base of the tail with 100 μL of an emulsion containing 100 μg of type-2 collagen and 250 μg of *M. tuberculosis*. The same injection was repeated on day 21.

### Assessment of arthritis

The macroscopic appearance of arthritis was observed up to day 70 after the first immunization. The severity of arthritis was scored in each of the four limbs per mouse on a scale of 0–4 as described previously [19]. The criteria for grading were: 0, no evidence of erythema and swelling; 1, erythema and mild swelling confined to the mid-foot or ankle joint; 2, erythema and mild swelling extending from the ankle to the mid-foot; 3, erythema and moderate swelling extending from the ankle to the metatarsal joints; 4, erythema and severe swelling encompass the ankle, foot, and digits. The maximum arthritis score was determined as the highest arthritis score in each mouse during the experimental period.

### Histological analysis

The excised joint was fixed with 10% formaldehyde. It was decalcified with Morse’s solution, and processed for routine paraffin embedding. Tissue cross-sections (5 μm) were stained with hematoxylin and eosin (HE) or safranin O in a standard manner.

### Recombinant mLRG preparation

A549, human alveolar adenocarcinoma cells, were transfected with pEBMulti-neo vector (Wako, Osaka, Japan) to obtain mouse LRG-expressing cells. Cells were cultured for 72 h in serum-free RPMI medium (Wako). LRG protein secreted into culture supernatant was purified with an antibody affinity column (NHS-activated Sepharose 4 Fast Flow conjugated with anti mLRG antibody mLRA0010) and concentrated by ultrafiltration (Amicon Ultra 10 K, Merk Millipore). Concentration of LRG was determined by mouse LRG ELISA as described subsequently.

### Quantitative real-time and direct reverse transcriptase PCR of messenger (m)RNA

Total RNA was isolated using the RNeasy Mini kit (Qiagen, Tokyo, Japan) according to the manufacturer’s protocol. First, 100 ng of RNA was reverse transcribed using the QuantiTect reverse transcription kit (Qiagen). For quantitative real-time reverse transcriptase PCR, standard curves for IL-17A, retinoid-related orphan receptor (ROR) γt, and hypoxanthine phosphoribosyl transferase 1 (HPRT1) were generated. Relative quantification of the PCR products was performed using ABI prism 7700 (Applied Biosystems, Darmstadt, Germany) and the comparative threshold cycle (CT) method. The level of the target gene expression was normalized to that of HPRT1 in each sample. The primers used for real-time PCR were as follows: *IL17A*, sense 5′-TCTCATCCAGCAAGAGATCC -3′, antisense 5′-GAATCTGCCTCTGAATCCAC -3′; *ROR*γ*t*, sense 5′-CCGCTGAGAGGGCTTCAC -3′, antisense 5′- TGCAGGAGTAGGCCACATTACA -3′; *HPRT1*, sense 5′-TCAGTCAACGGGGGACATAA-3′, antisense 5′-GGGGCTGTACTGCTTAACCAG-3′. Each reaction was performed in triplicate. The variation within samples was less than 10%.

### Western blot analysis

Whole-cell protein extract was prepared from CD4 cells in radioimmunoprecipitation assay (RIPA) buffer 10 mmol/L Tris-HCl (pH 7.5), 150 mmol/L NaCl, 1% (v/v) NP-40, 0.1% (w/v) SDS, 0.5% (w/v) sodium deoxycholate, 1% protease inhibitor cocktail (Nacalai Tesque, Tokyo, Japan), and 1% phosphatase inhibitor cocktail (Nacalai Tesque). The extracted proteins were resolved on SDS–PAGE and transferred to an Immnobilon-P transfer membrane (Millipore, Bedford, MA, USA). The following antibodies were used: anti-phospho-Smad2 (Ser465/467) (1:1000), anti-Smad2 (D43B4) (1:1,000), anti-phospho-Smad1 (Ser463/465), anti-Smad1(D59D7), anti-phospho-p38 (Thr180/Tyr182) (1:1,000), anti-p38 (#9211), anti-phospho-STAT3 (D3A7)/STAT3 (C-20) (1:1,000) (total STAT3 was from Santa Cruz, Santa Cruz Biotechnology, CA, USA. Other antibodies were from Cell Signaling Technology, Danvers, MA, USA); anti-glyceraldehyde-3-phosphate dehydrogenase (GAPDH) (1:2,000) (Santa Cruz Biotechnology). This was followed by treatment with 1:5000 diluted anti-rabbit horseradish-peroxidase-conjugated secondary antibodies (Cell Signaling Technology) and visualization using the Chemi-Lumi one L reagent (Nacalai Tesque). Band intensities were quantified with ImageJ 1.34.

### ELISA

The concentrations of IL-17, IL-6, IL-10, IL-21, Interferon gamma (IFN-γ),TNF-α (Biolegend, San Diego, CA, USA), soluble IL-6 receptor (IL6Ra) (R&D systems, Minneapolis, MN, USA) and anti-chicken type II collagen IgG antibody (Chondrex, inc., Redmond, WA, USA) in mouse sera on day 27 and cell culture supernatants were determined by ELISA according to the manufacturer’s protocol. The levels of serum LRG on day 27 were analyzed by sandwich ELISA using two antibody clones (mLRA0010 and rLRA0094) as described before [20].

### T cell differentiation

Naïve CD4^+^CD25^-^CD62L^+^ T cells were isolated from spleen and lymph nodes using FACS Aria (BD bioscience, San Jose, CA, USA). PerCPCy5.5-conjugated anti-CD4 (GK1.5), fluorescein isothiocyanate (FITC)-conjugated anti-CD25 (7D4), phycoerythrin (PE)-conjugated anti-CD62L (MEL-14) were purchased from Biolegend. The naïve T cells were cultured in DMEM (Wako, Osaka, Japan) supplemented with 10% fetal bovine serum (Biowest, Nuaillè, France) and penicillin G and streptomycin. Naïve T cells were activated with anti-CD3/CD28 beads (Life Technologies, Oslo, Norway), anti-IL-4 (10 μg/mL) (Biolegend), and anti-IFNγ (10 μg/mL) neutralizing antibodies (Biolegend) and were polarized with cytokines to generate Treg (TGF-β: 0.125 or 0.5 ng/mL, Peprotech, Inc, Rocky Hill, NJ, USA) or Th17 (TGF-β: 2 ng/mL and IL-6: 100 ng/mL, Peprotech, Inc) in the presence or absence of LRG. Cells were harvested on day 3 for analysis.

For determination of the differentiation of CD4+ T cells, inguinal lymphoid cells were isolated on day 27, and cultured in the presence of type-2 collagen (Chondrex,Inc. Redmond, WA, USA) for 3 days. Then, T cell subsets were analyzed by flow cytometry.

### Flow cytometry

For intracellular cytokine staining, cells were treated with 50 ng/mL phorbol-12-myristat-13-acetate (PMA) and 500 ng/mL ionomycin, in the presence of 3 μM goldi-stop (BD bioscience) for 4 h. Flow cytometric analysis of T cells was performed with PerCPCy5.5-conjugated anti-CD4 (GK1.5), allophycocyanin (APC)-conjugated anti-IL17 (TC11-18H10.1), PE-conjugated anti-IL6 receptor alpha (D7715A7), and PE-Cy7-conjugated anti-IFNγ (XMG1.2) from Biolegend (San Diego, CA, USA). eFlour450-conjugated anti-Foxp3 (FJK16S) and PE-conjugated gp130 (KGP130) were from eBioscience (San Diego, CA, USA).

Splenocytes were isolated and stimulated by IL-6 for 30 minutes for phosphorylated STAT3 analysis. These cells were fixed by 4% paraformaldehyde for 10 minutes at room temperature and permeabilized by ice-cold 90% methanol for 30 minutes. Cells were stained by Alexa Fluor-conjugated anti-phosphorylate STAT3 (py705, BD bioscience), PE-conjugated anti-CD25, and PerCPcy5.5-conjugated anti-CD4.

### Immunohistochemical analysis

Immunohistochemical analysis of joints was performed according to our previous report [20]. Briefly, 5-μm-thick paraffin sections were de-waxed, rehydrated and incubated for 16 h in citrate buffer (10 mM citric acid, pH6.0) at 60 °C for antigen retrieval. Sections were treated with 0.3% H2O2/MeOH, then blocked with Blocking One (Nacalai Tesque) and incubated with anti-LRG1 polyclonal antibody (clone R322, IBL, Gunma, Japan) overnight at 4 °C. After washing, the reaction was visualized by adding ABC solution followed by diaminobenzene (DAB) substrate (Vector Laboratories, Burlingame, CA, USA). All sections were counterstained with hematoxylin.

For naïve T cell immunocytochemical staining, isolated CD4^+^CD25^-^CD62L^+^ naïve T cells were placed onto a glass slide by cytospin. These cells were fixed by 4% paraformaldehyde for 10 minutes, blocked with Blocking One (Nacalai Tesque) for 1 h and then incubated with anti-IL-6 receptor monoclonal antibody (Bioss, Inc. Woburn, MA, USA) for 1 h at room temperature. The reaction was visualized by adding ABC solution followed by DAB substrate (Vector Laboratories, Burlingame, CA). All specimens were counterstained with hematoxylin.

### Statistical analysis

Statistical analysis of serum LRG was performed by one-way analysis of variance followed by Dunnett’s test. The assessment of arthritis was statistically analyzed using the Mann-Whitney *U* test. The levels of phosphorylated Smad2 relative to total Smad2 were analyzed by one-way analysis of variance followed by the Bonferroni test. Other statistical analyses were performed using the two-tailed Student’s *t* test. *P* < 0.05 was considered statistically significant.

## Results

### The symptoms of CIA were less severe in LRG KO mice compared to WT mice

**Fig. 1:**
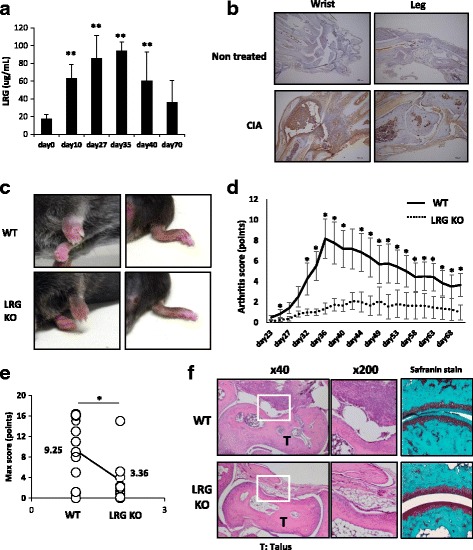
Leucine-rich alpha 2 glycoprotein (*LRG*) knockout (*KO*) mice are protected against collagen-induced arthritis (*CIA*). Male wild-type (*WT*) mice and LRG KO mice were subjected to CIA. **a** The serum levels of LRG in WT mice (*n* = 6–8) on indicated days after arthritis induction were determined by ELISA. Values are mean ± SD; **p < 0.01, Dunnett’s test. **b** Representative immunohistochemical analysis of LRG in wrist and ankle joints from non-treated WT mice or mice with CIA on day 35 after arthritis induction. **c** Representative macroscopic joint symptoms in WT mice or LRG KO mice on day 35 after arthritis induction. **d**, **e** The symptoms of arthritis were scored (0–4 per limb) until day 70. The arthritis score (**d**) and maximum arthritis score (**e**) are shown for WT mice (*n* = 12) and LRG KO mice (*n* = 11). Values are mean ± SD: *p < 0.05, Mann-Whitney *U* test. **f** Representative histological appearance of joints from a WT mouse and LRG KO mouse on day 35 after arthritis induction. Joints were stained with hematoxylin and eosin (*left* and *middle* panel) or safranin O (*right* panel)

At first, we determined the serum levels of LRG in the CIA model. In WT mice with CIA, serum LRG levels increased after the first immunization, peaked at around day 35 and remained significantly high until day 40 (Fig. 1a). On immunohistochemical analysis, LRG was intensively stained in the joints of WT mice with CIA, but not in non-treated WT mice (Fig. 1b), indicating that LRG expression is upregulated in arthritic joints. These results suggest that LRG may contribute to the pathogenesis of this model. To examine the functional role of LRG in the CIA model, WT and LRG KO mice were subjected to CIA. WT mice developed signs of arthritis after the second collagen immunization and swelling of the joint reached a peak around day 35. On the contrary, the symptoms of arthritis in LRG KO mice were mild and the arthritis score in KO mice was significantly lower than that in WT mice (Fig. 1c and d). Moreover, the maximum score in LRG KO mice was also significantly lower than that in WT mice (Fig. 1e). On histological examination of the ankle joints in WT mice on day 35 there was noticeable hyperplasia of synovial tissue and bone erosion due to inflammatory cell infiltration, whereas this was not observed in LRG KO mice (left and middle panels of Fig. 1f). Safranin O staining revealed a loss of cartilage surface in the arthritic joints in WT mice. In contrast, LRG deficiency protected mice from the cartilage destruction (right panel of Fig. 1e). These results indicate that LRG plays an important role in the development of joint inflammation in the CIA model.

### Th17 differentiation, but not Treg induction was inhibited in LRG KO mice with CIA

**Fig. 2:**
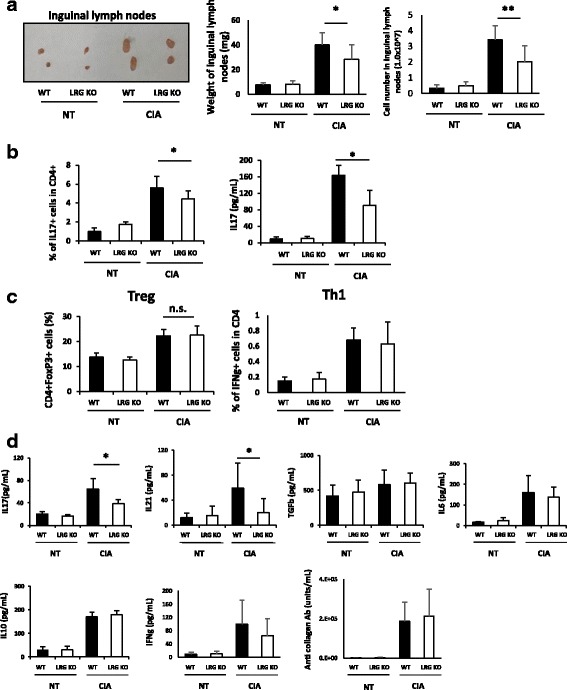
Leucine-rich alpha 2 glycoprotein (LRG) deficiency results in impaired differentiation of T helper (Th)-17 cells after induction of arthritis. **a** Representative macroscopic images (*left*), the weight (*middle*) and the cell number (*right*) of inguinal lymph nodes in wild-type (*WT*) mice (*n* = 16) and LRG knockout (*LRG KO*) mice (*n* = 16) with or without arthritis induction (day 27): *p < 0.05. **b** Inguinal lymph node cells on day 27 were isolated from WT mice (*n* = 8) and LRG KO mice (*n* = 8) and stimulated by chicken type-2 collagen for 3 days. The percentages of IL-17+ cells gated on CD4+ cells were determined by flow cytometry (*left*). The levels of IL-17 in cell culture supernatants were analyzed by ELISA (*right*): *p < 0.05. **c** Percentages of CD4 + Foxp3 + cells (*Treg*) and CD4 + IFN-γ + cells (Th1) of the same population as **b** were determined by flow cytometry. **d** The serum levels of cytokines and anti-collagen type-2 antibodies in WT mice (n = 8) and LRG KO mice (n = 8) on day 27 after induction of collagen-induced arthritis (*CIA*) were analyzed by ELISA: *p < 0.05. All statistical analyses were performed using Student’s *t* test. *NT* non-treated, *n.s.* not significant, *IFN* interferon

During adaptive immune response, naïve CD4 T cells are activated and differentiated initially in lymphoid tissues and then migrate into local inflammatory sites. Accordingly, collagen immunization in WT mice and LRG KO mice induced enlargement of inguinal lymph nodes prior to joint inflammation (Fig. 2a, left). However, the weight of the inguinal lymph nodes was significantly lower in LRG KO mice than in WT mice (Fig. 2a, middle). The cell number in inguinal lymph nodes was also decreased in LRG KO mice compared to WT mice (Fig. 2a, right). To examine the role of LRG in the initial adaptive immune response, we next evaluated the helper T cell differentiation in inguinal lymph nodes on day 27. Inguinal lymph node cells from WT or LRG KO mice were cultured in the presence of chicken type-2 collagen to analyze the response of T helper subsets against type-2 collagen. There were significantly fewer Th17 cells retrieved after collagen stimulation in LRG KO mice than in WT mice and cells from KO mice produced less IL-17 than those from WT mice (Fig. 2b). In contrast, there were no significant differences in the size of the Treg and Th1 populations in WT mice and LRG KO mice (Fig. 2c). The serum levels of IL-17 and IL-21, which are produced mainly by Th17 cells, were significantly lower in LRG KO mice than in WT mice, but the levels of IL-6 and TGF-β, which have critical roles in Th17 differentiation, were not different in these mice. In addition, there were no significant differences in IFN-γ or IL-10, which are produced mainly by Th1 and Treg cells, respectively, or in anti-collagen type-2 antibodies, which are produced by B lineage cells. These results suggest that LRG deficiency leads to attenuated immune response, characterized by the suppression of Th17 differentiation in the CIA model.

### LRG enhanced the TGF-β-induced Smad2 phosphorylation and had distinct effects on naïve T cell differentiation depending on surrounding cytokines

**Fig. 3:**
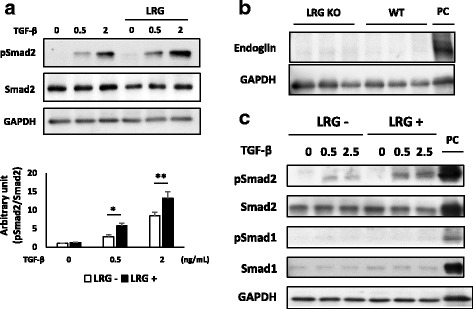
Recombinant leucine-rich alpha 2 glycoprotein (*LRG*) enhances transforming growth factor beta (*TGF-*β)-induced Smad2 phosphorylation in naïve CD4 T cells and promotes T regulatory cell differentiation. **a** Naïve CD4 T cells were isolated from LRG knockout (*LRG KO*) mice and were stimulated by TGF-β (0.5 or 2 ng/mL) in the presence or absence of recombinant LRG (*LRG +* (1 μg/mL) or *LRG -*) for 30 minutes. Representative data from three independent experiments are shown (*upper panel*). The levels of phosphorylated Smad2 relative to total Smad2 were quantified using imageJ and values (mean ± SD; *n* =3) are shown (*bottom panel*): *p < 0.05, **p < 0.01, one-way analysis of variance followed by the Bonferroni test. *NT* non-treated. **b** Naïve CD4 T cells were isolated from wild-type (*WT*) or LRG KO mice (*n* = 3, respectively). The expression of endoglin was determined by western blotting. MS-1, mouse endothelial cell, was used as a positive control (*PC*) for the expression of endoglin. **c** Naïve CD4 T cells were obtained from LRG KO mice and stimulated by TGF-β (0, 0.5 and 2.5 ng/mL) in the absence or presence of recombinant LRG (*LRG +* (1 μg/mL) or *LRG -*) for 30 minutes. The phosphorylation of Smad1 and Smad2 were examined by western blotting. L929, mouse fibroblast, was used as a PC. *GAPDH* glyceraldehyde-3-phosphate dehydrogenase

TGF-β is one of the key cytokines that regulate T helper cell differentiation. In addition, we previously demonstrated that LRG enhances TGF-β-induced Smad2 phosphorylation in several cancer cell lines [4]. To examine the effect of exogenous LRG on TGF-β signaling in CD4 T cells, we stimulated naïve CD4 T cells derived from LRG KO mice with TGF-β in the absence or presence of recombinant LRG. As shown in Fig. 3a, TGF-β phosphorylated Smad2 in a dose-dependent manner, and recombinant LRG significantly enhanced this effect.

A previous study showed that LRG binds to endoglin, a co-receptor of the TGF-β superfamily, and enhances the TGF-β-induced phosphorylation of Smad1/5 rather than Smad2 in endothelial cells [3]. Given that not all cell types express endoglin, we then examined the expression of endoglin in naïve CD4 T cells isolated from WT or LRG KO mice. As shown in Fig. 3b, endoglin protein was detected in the control endothelial cells, whereas there was no detectable expression of endoglin in naïve CD4 T cells in either WT or LRG KO mice. Next, we examined the phosphorylation of Smad1 and Smad2 in naïve CD4 T cells isolated from LRG KO in the absence or presence of recombinant LRG. Smad2 in naïve CD4 T cells was phosphorylated by stimulation of TGF-β. In contrast, the phosphorylation of Smad1 was not observed even in the presence of recombinant LRG (Fig. 3c). Our results suggest that endoglin is not expressed in naïve CD4 T cells and LRG predominantly stimulates the TGF-β-Smad2 signaling pathway in these cells.

**Fig. 4:**
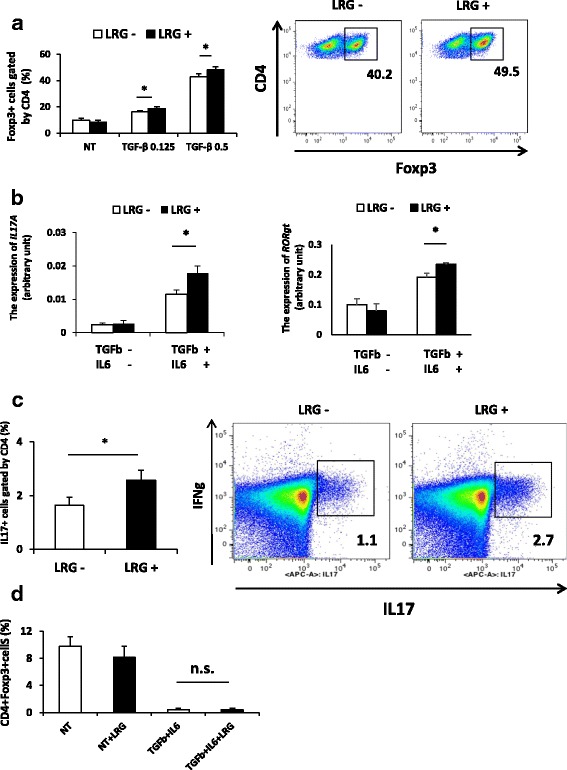
Recombinant leucine-rich alpha 2 glycoprotein (*LRG*) enhances differentiation of T helper 17 (Th17) cells under the Th17 polarization condition. **a** Naïve CD4 T cells (*n* = 3) were treated for 3 days with anti-CD3/CD28 beads and transforming growth factor beta (*TGF-*β) (0, 0.125 or 0.5 ng/mL (Treg polarization condition)) in the presence or absence of LRG (*LRG +* (1 μg/mL) or *LRG -*). The percentage of CD4^+^Foxp3^+^ T regulatory (Treg) cells was determined by flow cytometry (*right*): *p < 0.05. Representative data are shown (*left panel*). **b**, **c**, **d** Naïve CD4 T cells were isolated from the spleen and lymph nodes of LRG knockout mice (*LRG KO*) (*n* = 3) and cultured for 3 days with anti-CD3/CD28 beads in the presence or absence of recombinant LRG (*LRG +* (1 μg/mL) or *LRG -*) under a non-polarization condition without cytokines or the Th17 polarization condition with TGF-β (2 ng/mL) and IL-6 (100 ng/mL). **b** The expression of IL-17 (*left*) and RORγt (*right*) mRNA was analyzed by quantitative PCR: *p < 0.05. **c** The percentage of IL-17+ cells in the CD4+ population under the Th17 polarization condition was determined by flow cytometry. Values are mean ± SD, n = 3 (*left panel*). Representative data are shown (*right panel*). **d** The induction of Treg cells under a non-polarization condition or under the Th17 polarization condition was examined by flow cytometry. All statistical analyses were performed using Student’s *t* test. NT; non-treated

Because TGF-β-Smad2 signaling is an essential pathway for the differentiation of both Treg and Th17 populations [15–17], we next investigated the effect of LRG in T helper differentiation in vitro. Under the Treg polarization condition, recombinant LRG increased the induction of CD4^+^Foxp3^+^ Treg (Fig. 4a). On the other hand, under Th17 polarization condition, LRG enhanced the expression of Th17-related genes such as IL-17A and RORγt (Fig. 4b) and increased Th17 population (Fig. 4c) with no apparent influence on Treg differentiation (Fig. 4d). These results suggest that LRG enhances Treg differentiation when CD4 T cells are polarized by TGF-β alone but promotes Th17 differentiation when both TGF-β and IL-6 are present.

### LRG contributes to IL6-STAT3 signaling by upregulating the expression of IL6 receptor

**Fig. 5:**
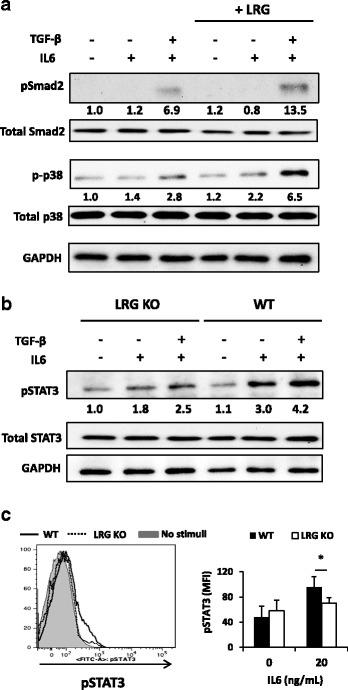
Leucine-rich alpha 2 glycoprotein (*LRG*) contributes to the IL-6-STAT3 signaling in naïve CD4 T cells by upregulating IL-6 receptor expression. **a** Naïve CD4 T cells were isolated from wild-type (*WT*) mice and stimulated by IL-6 (100 ng/mL) or IL-6 with transforming growth factor beta (*TGF-*β) (2 ng/mL) in the absence or presence of recombinant LRG (1 μg/mL) for 30 minutes. Phosphorylation of Smad2 and p38 was determined by western blotting. Relative band intensities of pSmad2 and p-p38, as normalized by Smad2 and p38, respectively, are depicted at the bottom of the bands. **b** Naïve CD4 T cells were isolated from WT or LRG knockout (*LRG KO*) mice and stimulated by IL-6 (100 ng/mL) in the absence or presence of TGF-β (2 ng/mL) for 30 minutes. STAT3 phosphorylation was determined by western blotting. Relative band intensities of pSTAT3 normalized by STAT3 are depicted at the bottom of the bands. **c** Splenocytes were isolated from WT and LRG KO mice (*n* = 3 for each group) and stimulated by IL-6 (100 ng/mL) for 30 minutes. After intracellular staining, phosphorylated STAT3 in CD4 + CD62 + CD25 - cells was determined by flow cytometry. Representative data are shown in the *left panel* and mean fluorescence intensity of phosphorylated STAT3 (mean ± SD) is shown in the *right panel*: *p < 0.05. *GAPDH* glyceraldehyde-3-phosphate dehydrogenase

Our in vitro experiments suggest that LRG is involved in the differentiation of both Treg and Th17 cells, but our analysis on the CIA model underscored a part played by LRG in Th17 differentiation. We first examined whether LRG enhanced TGF-β signaling even under the Th17 polarization condition Similar to the results of the Treg polarization condition, LRG enhanced Smad2 phosphorylation under Th17 polarization. In addition, p38 phosphorylation, which is Smad-independent downstream of TGF-β signaling, was also increased by the addition of recombinant LRG (Fig. 5a). These findings indicated that LRG promotes TGF-β signaling by enhancing activation of both Smad-dependent and Smad-independent pathways. To explore further the contribution of LRG on Th17 generation, we next evaluated the effect of LRG on IL-6 signaling, which plays pivotal roles in promoting Th17 differentiation. Interestingly, western blotting revealed that upon stimulation with IL-6 alone or together with TGF-β, STAT3 phosphorylation was attenuated in naïve CD4 T cells derived from LRG KO mice compared to WT mice (Fig. 5b). Similar data were obtained by flow cytometric analysis of phosphorylated STAT3 (Fig. 5c). These results suggest that LRG deficiency results in attenuated IL-6 signaling, which might account for the suppression of Th17 differentiation in CIA.

**Fig. 6:**
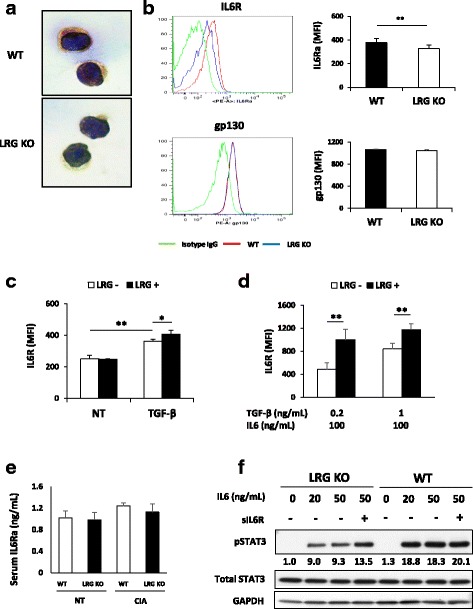
Leucine-rich alpha 2 glycoprotein (*LRG*) upregulates the expression of IL-6 receptor (*IL-6R*) in naïve CD4 T cells presumably through the enhancement of transforming growth factor beta (*TGF-*β) signaling. **a**, **b** Naïve CD4 T cells were isolated from the spleen and lymph nodes of wild-type (*WT*) or LRG knockout (*LRG KO*) mice (n = 8 in each group) and the expression of IL-6R and gp130 were examined. **a** Representative data from immunocytochemical analysis of IL-6R. **b** The expression of IL-6R or gp130 in naïve CD4 T cells was analyzed by flow cytometry and representative data are shown in the *left panel*. Mean values of mean fluorescence intensity (*MFI*) ± SD are shown in the *right panel*: **p < 0.01. **c**, **d** Naïve CD4 T cells obtained from LRG KO mice (*n* = 3) were stimulated by TGF-β (1 ng/mL) (**c**) or TGF-β (0.2 or 1 ng/mL) with IL-6 (100 ng/mL) (**d**). After 24 h of culture, IL-6R expression was analyzed by flow cytometry. Values are mean ± SD: *p < 0.05. **e** The serum levels of soluble IL-6 receptor alpha (*IL-6Ra*) of WT or LRG KO mice on day 27 of CIA were determined by ELISA. **f** Naïve CD4 T cells derived from WT and LRG KO mice were stimulated by IL-6 (20 or 50 ng/ mL) in the presence or absence of soluble IL-6R (20 ng/mL) for 30 minutes. STAT3 phosphorylation was determined by western blotting. Relative band intensities of pSTAT3 normalized by STAT3 are depicted at the *bottom* of the bands. *NT* non-treated

A previous study showed that STAT3 phosphorylation in CD4 T cells was increased and sustained in the presence of both TGF-β and IL-6, as compared with IL-6 alone [21]. In addition, it was reported that Smad2 enhances Th17 differentiation in part by upregulating the expression of IL-6 receptor α chain (IL-6R) on CD4 T cells [17]. These observations raise the possibility that LRG, by enhancing TGF-β-Smad2 signaling, may contribute to IL-6 signaling by regulating the expression of IL-6R. Therefore, we examined the expression of IL-6R in CD4 T cells from WT or LRG KO mice. The immunocytochemical (Fig. 6a) and flow cytometric analysis (Fig. 6b) revealed that IL-6R expression on naïve CD4 T cells was significantly reduced in LRG KO mice compared to WT mice. Interestingly, LRG deficiency had no effect on gp130 expression, a signal transducing subunit of the IL-6R complex (Fig. 6b). In addition, consistent with the previous finding [17], TGF-β increased the expression of IL-6R in CD4 T cells and this effect was promoted by the addition of LRG (Fig. 6c). Moreover, LRG further enhanced the expression of IL-6R 24 h after Th17-polarizing stimulation (Fig. 6d). Next, we examined the levels of serum-soluble IL-6R (sIL-6R). Like IL-6R on the cell surface [22], sIL-6R binds to IL-6 and can stimulate cells via the signal-transducing protein gp130 [23, 24]. However, serum sIL-6R levels were similar in WT and LRG KO mice and they did not increase after collagen challenge, suggesting that the effect of LRG on IL-6R is specific to the membrane-bound form of IL-6R in CD4 T cells. To determine whether impaired STAT3 phosphorylation is caused by the reduction in IL-6R expression, we stimulated naïve CD4 T cells by IL-6 in the presence of sIL-6R. In WT naïve CD4 T cells, which express endogenous IL-6R, the phosphorylation of STAT3 was not enhanced by sIL-6R (Fig. 6d). However, in naïve T cells from LRG KO mice, the diminished STAT3 phosphorylation was markedly restored by the addition of sIL-6R (Fig. 6d). These results suggest that LRG deficiency attenuates IL-6/STAT3 signaling in CD4 T cells by modulating IL-6R expression, resulting in impaired Th17 response.

## Discussion

We previously reported that LRG binds with TGF-β and modulates the TGF-β-induced smad2 pathway [4]. Consistent with this observation, we confirmed that LRG enhanced the phosphorylation of Smad2 in naive CD4 T cells in this study. Accumulating evidence indicates that the Smad2 pathway plays important but complicated roles in naïve CD4 T cell differentiation. Smad2 phosphorylation induces the expression of Foxp3, which promotes Treg differentiation and immune suppression by interfering with RORγt, a critical transcriptional factor of Th17 [16]. Smad2, on the other hand, also acts as a positive regulator of Th17, which plays critical roles in chronic inflammation including in RA [25, 26]. By mediating TGF-β-induced IL-6R expression [17], Smad2 can enhance IL-6-STAT3 signaling, which represses the function of Foxp3 and initiates Th17 differentiation [9, 27]. Thus, it is likely that Smad2 directs the differentiation of naïve CD4 T cells toward Treg or Th17, depending on the absence or presence of IL6, respectively. Thus, LRG, as an enhancer of Smad2 activation, can regulate Treg and Th17 differentiation dependently on the cytokine milieu.

IL-6 is a pro-inflammatory cytokine that is critically involved in the pathogenesis of RA. Levels of IL-6 in both serum and synovial fluid are elevated in RA patients and are associated with disease activity [28, 29]. Moreover, anti-IL-6 receptor antibody treatment induces significant amelioration in the clinical symptoms of arthritis, accompanied by a decrease in Th17 cells [30]. In the present study, we confirmed the elevation of IL-6 in the active stage of CIA. In addition, our findings indicate that LRG promotes the differentiation of Th17 rather than Treg in vitro when both TGF-β and IL-6 are present. Thus, in the CIA model and probably in RA in which excessive IL-6 production is detectable, LRG likely enhances Th17 differentiation and promotes joint inflammation by augmenting Smad2 activation. This notion is consistent with the previous findings that T-cell-specific deficiency of Smad2 leads to impaired Th17 differentiation and alleviated clinical symptoms in mouse disease models including experimental autoimmune encephalomyelitis and CIA [16, 31].

Interestingly, high levels of IL-6R are detectable in naïve CD4 T cells, but the expression is diminished during inflammation [32]. In the pathogenesis of arthritis, it is likely that IL-6 signals are particularly important for the initial priming of CD4+ T cells, because our group previously revealed that anti-IL-6R antibody treatment on day 0 of CIA inhibited both Th17 induction and arthritis, but administration on day 14 had no effect [33]. In addition, Nish et al. recently reported that T-cell-specific ablation of IL-6R was sufficient to abrogate Th17 differentiation [33]. These findings collectively suggest that IL-6R expression in naïve CD4 T cells is critically important for the initial stage of Th17 differentiation. Our data showed that the expression of IL-6R is reduced in naïve CD4 T cells in LRG KO mice. Moreover, in this study, we observed that LRG increases the expression of IL-6R in CD4 T cells after TGF-β stimulation and even more after Th17 priming. Thus, LRG can support Th17 differentiation by maintaining IL-6R expression in naïve CD4 T cells. In addition to this, LRG might enhance the Th17 cell differentiation via p38 signaling, given that the p38 pathway regulates the differentiation of Th17 cells [34].

Besides the role in Th17 cell differentiation, IL-6 signal is reported to be involved in the survival of CD4 T cells [35]. In addition, T-cell-specific deletion of IL-6R causes a defect in T cell proliferation [36]. Our study showed the enlargement of inguinal lymph nodes was suppressed in LRG KO mice compared with WT mice. One possible reason for this is that LRG deficiency might lead to enhanced apoptosis and impaired proliferation of CD4 T cells due to reduced IL-6R expression. Furthermore, we revealed that LRG could enhance the phosphorylation of p38, a crucial pathway of cell proliferation and survival. Therefore, decreased p38 signaling in CD4 T cells might also contribute to the attenuated lymph node response in LRG KO mice.

Our data indicate that LRG promotes Treg polarization in vitro. However, whereas LRG deficiency resulted in a reduction in Th17 cells during CIA in vivo, the frequency of Tregs was not affected either in non-treated or collagen-immunized mice. This may be due to the difference between Treg and Th17 cells in TGF-β dependency. Under the normal condition, the majority of Treg cells are thymus-derived Foxp3+ Treg cells. Although TGF-β is critical for Foxp3 induction in naïve CD4 T cells in the periphery, it is less important in the development of thymus-derived Treg cells [37]. We therefore consider that thymus-derived Treg cells might mask a defect in TGF-β-induced Treg development due to LRG deficiency.

In this study, we showed that arthritis in LRG KO mice was significantly reduced at the onset of the symptoms. We then determined the influence of LRG on the initiation of adaptive immune responses, focusing on the differentiation of naïve CD4 T cells. However, taking into consideration the remarkable suppression of arthritis in LRG KO mice throughout the course of the disease, LRG might play other important roles in the pathogenesis of arthritis. To examine a possible defect in humoral immune response in LRG KO mice, we measured anti-collagen antibodies. However, these antibody levels were similar in LRG KO and WT mice, suggesting that the humoral immune response to collagen is not altered in LRG KO mice. Previous reports showed that LRG is expressed in neutrophils, co-localized with myeloperoxidase in their granules and involved in their differentiation and activation [38, 39]. Interestingly, the gene for human LRG localizes to chromosome 19p13.3, where the genes for primary neutrophil granule enzymes also localizes [39]. In addition, because IL-17 produced by Th17 recruits neutrophils by inducing neutrophil chemoattractants [40], LRG may indirectly enhance neutrophil migration into the joints. Given that activated neutrophils are found in synovial tissue in RA and play critical roles in joint destruction [41], LRG may enhance arthritis by regulating neutrophil development, recruitment and function. Furthermore, previous studies showed that LRG can promote angiogenesis in damaged tissue [3, 5] by modulating TGF-β signaling in endothelial cells. In RA, hypoxia in synovium likely induces synovial angiogenesis [42], which pivotally contributes to the pathogenesis of disease in the joints [43]. Hypoxia-inducible factor (HIF)1-α, which is highly expressed in the synovium in RA, regulates the expression of pro-angiogenic mediators including endoglin [44]. In addition, LRG is abundantly expressed in inflammatory lesions in CIA and other diseases [20, 45], where the expression is likely mediated by various inflammatory cytokines such as IL-6, IL-22, TNFα, and IL-1β [1]. It is suggested that LRG binds with endoglin in endothelial cells and promotes angiogenesis by enhancing pro-angiogenic Smad1/5/8 signaling of TGF-β [3]. Therefore, LRG might facilitate intracapsular inflammation by orchestrating many cell types such as CD4 T cells, neutrophils and endothelial cells.

## Conclusion

Our study indicates that LRG promotes the differentiation and the proliferation of Th17 in vivo and contributes to the development of CIA through enhancement of TGF-β-Smad2 signaling. While LRG has been highlighted as a potential biomarker of active RA, LRG might also emerge as a new therapeutic target. However, considering that TGF-β is a pleiotropic cytokine with contradictory functions, LRG might exert distinct effects in other pathophysiological conditions, especially in the absence of IL-6. Future study on other disease models will be needed to address this issue.

## Abbreviations

CIA: Collagen induced arthritis
DMEM: Dulbecco’s modified Eagle’s medium
ELISA: Enzyme-linked immunosorbent assay
Foxp3: Forkhead box p3
GAPDH: Glyceraldehyde-3-phosphate dehydrogenase
HPRT1: Hypoxanthine phosphoribosyl transferase 1
IFN-γ: Interferon gamma
IL: Interleukin
KO: Knockout
LRG: Leucine-rich alpha 2 glycoprotein
PE: Phycoerythrin
RORγt: Retinoid-related orphan receptor γt
TGF-β: Transforming growth factor beta
Th17: T helper 17 cells
TNF-α: Tumor necrosis factor alpha
Treg: Regulatory T cells
WT: Wild-type
